# Real-Time Detection of a Self-Replicating RNA Enzyme

**DOI:** 10.3390/molecules21101310

**Published:** 2016-09-30

**Authors:** Charles Olea, Gerald F. Joyce

**Affiliations:** Department of Chemistry and The Skaggs Institute for Chemical Biology, The Scripps Research Institute, 10550 N. Torrey Pines Road, La Jolla, CA 92037, USA; colea@scripps.edu

**Keywords:** aptazyme, exponential amplification, fluorescence detection, in vitro selection, RNA enzyme, RNA ligation, self-replication

## Abstract

A system was developed to detect the self-replication of an RNA enzyme in real time. The enzyme is an RNA ligase that undergoes exponential amplification at a constant temperature and can be made to operate in a ligand-dependent manner. The real-time system is based on a fluorimetric readout that directly couples the ligation event to an increase in florescence signal that can be monitored using standard instrumentation. The real-time system can also operate entirely with l-RNA, which is not susceptible to degradation by ribonucleases that are present in biological samples. The system is analogous to real-time PCR, but with the potential to detect small molecules, proteins, and other targets that can be recognized by a suitable aptamer. The ligand-dependent self-replication of RNA has potential applications in molecular diagnostics and biosensing that benefit from the rapid, precise, and real-time detection of various target molecules.

## 1. Introduction

Technologies based on the exponential amplification of nucleic acids are widely used in molecular diagnostics and biosensing. For example, the quantitative polymerase chain reaction (qPCR) is a sensitive and precise method for detecting target nucleic acids [1,2]. Due to its capacity for exponential amplification, qPCR can detect minute amounts of a target. The starting concentration of the target can be determined based on the number of thermal cycles required to reach an amplification threshold. Other methods can be used to detect non-nucleic-acid targets based on the binding of the target to an antibody, aptamer, or other capture reagent. In such cases the binding event must usually be coupled to a means for signal amplification, such as enzyme turnover or nucleic acid amplification. A different approach is to employ a capture reagent that itself undergoes exponential amplification upon binding the target. Such a system has been demonstrated based on aptamer-containing, self-replicating RNA enzymes [3,4]. A major shortcoming of that system is that, unlike qPCR, it does not provide quantitation throughout the course of amplification. That shortcoming has now been addressed, enabling the real-time, ligand-dependent, exponential amplification of RNA.

**Figure 1:**
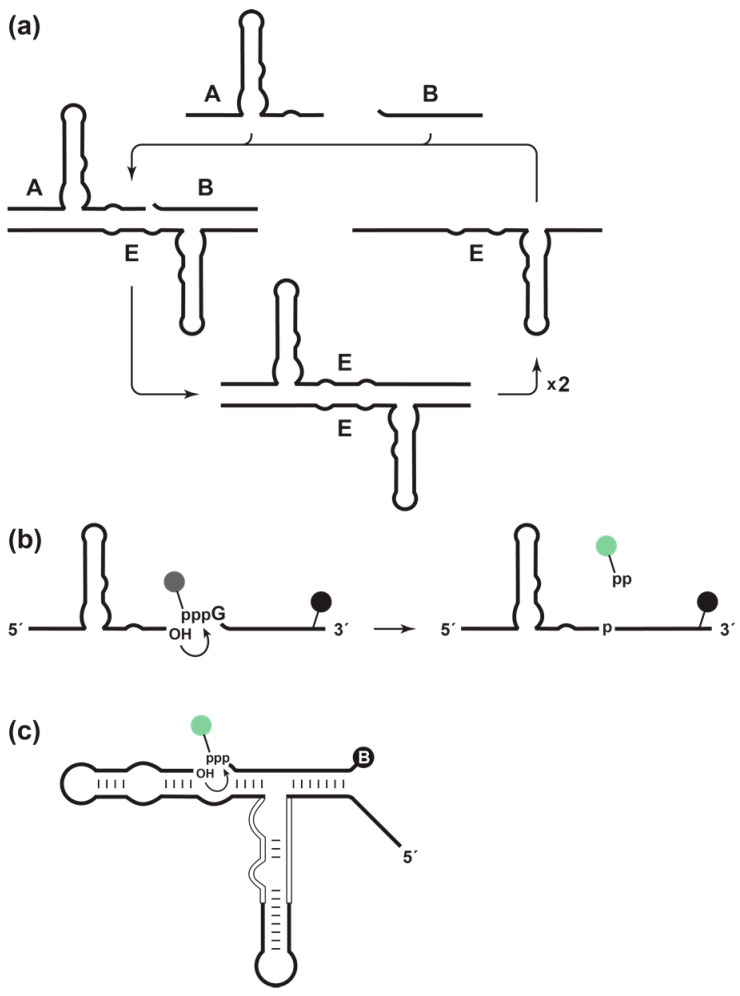
Amplification and detection of a self-replicating RNA enzyme. (**a**) The enzyme (E) catalyzes ligation of substrates A and B to form a new copy of E. The E•E complex then dissociates to begin the next replication cycle; (**b**) For real-time fluorescence detection, substrate B contains both a fluorophore linked to the 5′-γ-phosphate and a quencher at the 3′-end (shaded circles). Upon ligation, the fluorophore is released and no longer quenched; (**c**) The enzyme was evolved to accept 5′-fluorophore-labeled substrate B by introducing random mutations throughout its catalytic center (open lines), joining the 5′-end of substrate A to the 3′-end of E, and selecting molecules that catalyze ligation of 3′-biotinylated substrate B through their capture on streptavidin.

The self-replicating RNA enzyme (E) is an RNA ligase that catalyzes the joining of two RNA substrates (A and B) to form another copy of itself [5,6]. The ligation reaction involves an attack of the 3′-OH of substrate A on the 5′-triphosphate of substrate B, forming a 3′,5′-phosphodiester linkage and releasing inorganic pyrophosphate. The resulting E•E complex dissociates in a non-rate-limiting manner [7] to yield two copies of E, each of which can enter another replication cycle (Figure 1a). Replication continues at a constant temperature until the supply of substrates is exhausted. The rate of exponential amplification depends on the inherent catalytic properties of the enzyme. For the original self-replicating enzyme, this rate is 0.03 min^–1^, corresponding to a doubling time of 23 min [6]. For an improved form of the enzyme, the exponential amplification rate is 0.14 min^–1^, corresponding to a doubling time of 5 min [8].

The catalytic activity of the RNA enzyme can be made dependent on a target ligand by linking a ligand-binding aptamer domain to the catalytic center of the enzyme [3]. The resulting “aptazyme” [9] binds the ligand in a manner that stabilizes the catalytic center, thereby triggering enzymatic activity and causing exponential amplification to be dependent on the presence of the ligand. The concentration of the ligand relative to the *K*_d_ of the aptamer domain determines the degree of saturation of the aptazyme, which, as previously shown [3,4], determines the rate of exponential amplification relative to the rate at saturation. The faster the rate of amplification, the more quickly the system reaches a defined amplification threshold. Thus, like qPCR, the system can be used to measure precisely the concentration of the target ligand; however, unlike qPCR, it can be generalized to any target for which a suitable aptamer is available.

A previous limitation of the RNA replication system was that it could not be applied to biological samples without first removing ribonucleases that would otherwise degrade the RNA. This limitation was overcome by preparing the enzyme and its substrates in the form of enantiomeric l-RNA [10], which is impervious to ribonucleases. The d- and l-RNA enzymes have identical biochemical properties, but only the latter have been shown to operate in the presence of biological samples such as human serum [10]. This is important, for example, if one wants to measure the concentration of serum proteins.

For the ligand-dependent exponential amplification of RNA to be a practical method, it would be important to track the course of amplification in real time, based on the development of a fluorescent signal or other simple readout. Previous analyses were conducted after the reaction had been completed, using either polyacrylamide gel electrophoresis (PAGE) to determine the amount of ligated products or a luciferase assay that is driven by the release of inorganic pyrophosphate [3].

In this study, a fluorimetric method was developed to monitor the amplification of the self-replicating RNA enzyme in real time. The B substrate was labeled with a fluorophore linked to the γ-phosphate of the 5′-triphosphate, and a quencher was placed at the 3′-end (Figure 1b). Prior to ligation, fluorescence is quenched; however, upon ligation, the pyrophosphate is released together with the fluorophore, separating the fluorophore from the quencher and giving rise to a fluorescent signal. This is analogous to “pyrosequencing” methods for DNA sequencing, which employ modified dNTPs that contains both a fluorophore linked to the γ-phosphate and a quencher linked to the base [11]. Just as pyrosequencing required engineering the DNA polymerase to accept the modified dNTPs, it was necessary to engineer the self-replicating RNA enzyme to accept the modified B substrate. This was achieved using in vitro selection, which resulted in a variant form of the enzyme that can undergo exponential amplification with the modified substrate, can function as either d- or l-RNA, and can be made to operate in a ligand-dependent manner.

## 2. Results

### 2.1. In Vitro Selection

The original form of the self-replicating RNA enzyme [6] was tested using a B substrate with 5(6)-carboxytetramethylrhodamine (TMR) attached to the 5′-γ-phosphate. This substrate was prepared by in vitro transcription in a mixture containing γ-(6-aminohexyl)-GTP, then reacting with TMR *N*-hydroxysuccinimide (NHS) ester, followed by gel purification the conjugated product. The TMR-labeled substrate was tested in a self-replication reaction employing 19 µM of substrate A, 25 µM of TMR-B, 1.5 µM of E, and 25 mM of MgCl_2_ at pH 8.5 and 42 °C. The enzyme performed very poorly with the modified substrate, reaching only 7% completion after 25 h and failing to undergo exponential amplification. Similarly, poor performance was seen when other fluorophores, such as Cy5 or Alexa Fluor 488, were linked to the 5′-γ-phosphate of substrate B (data not shown).

In vitro selection was used to develop a modified form of the enzyme that can readily accept fluorophore-modified substrates. The selection strategy was based on a previous design [8] that requires ligation in a bimolecular format while maintaining key features of the self-replication reaction (Figure 1c). The 3′-end of the enzyme was connected to the 5′-end of substrate A via a stem-loop structure. The TMR-B substrate was modified at its 3′-end with biotin by first incorporating 2′-azido-modified deoxyuridine using poly(A) polymerase, then reacting with alkyne-modified biotin using click chemistry. Enzymes that were capable of ligating the fluorophore-modified substrate B to the attached substrate A were captured by binding to streptavidin, then selectively amplified by RT-PCR and subsequent in vitro transcription.

A population of enzyme variants was generated by randomizing 16 nucleotides that comprise the catalytic center (Figure 1c). A starting population of 6 × 10^12^ RNA molecules (100 pmol) was used to initiate the first round of selection. The first round employed 2 µM of TMR-B-biotin, 1 µM of E-A, and 25 mM of MgCl_2_, which were incubated at pH 8.5 and 42 °C for 1 h. During subsequent rounds, the selection pressure was increased by decreasing the concentration of reactants, decreasing the concentration of MgCl_2_, and decreasing the reaction time. By the fifth round, the reaction employed 50 nM of TMR-B-biotin, 50 nM of E-A, and 5 mM of MgCl_2_, which were incubated for only 15 s. The activity of the population was assayed after each round of selection and, by the fifth round, had reached a plateau, signifying that the process was completed.

### 2.2. Fluorophore-Compatible Enzyme

**Figure 2:**
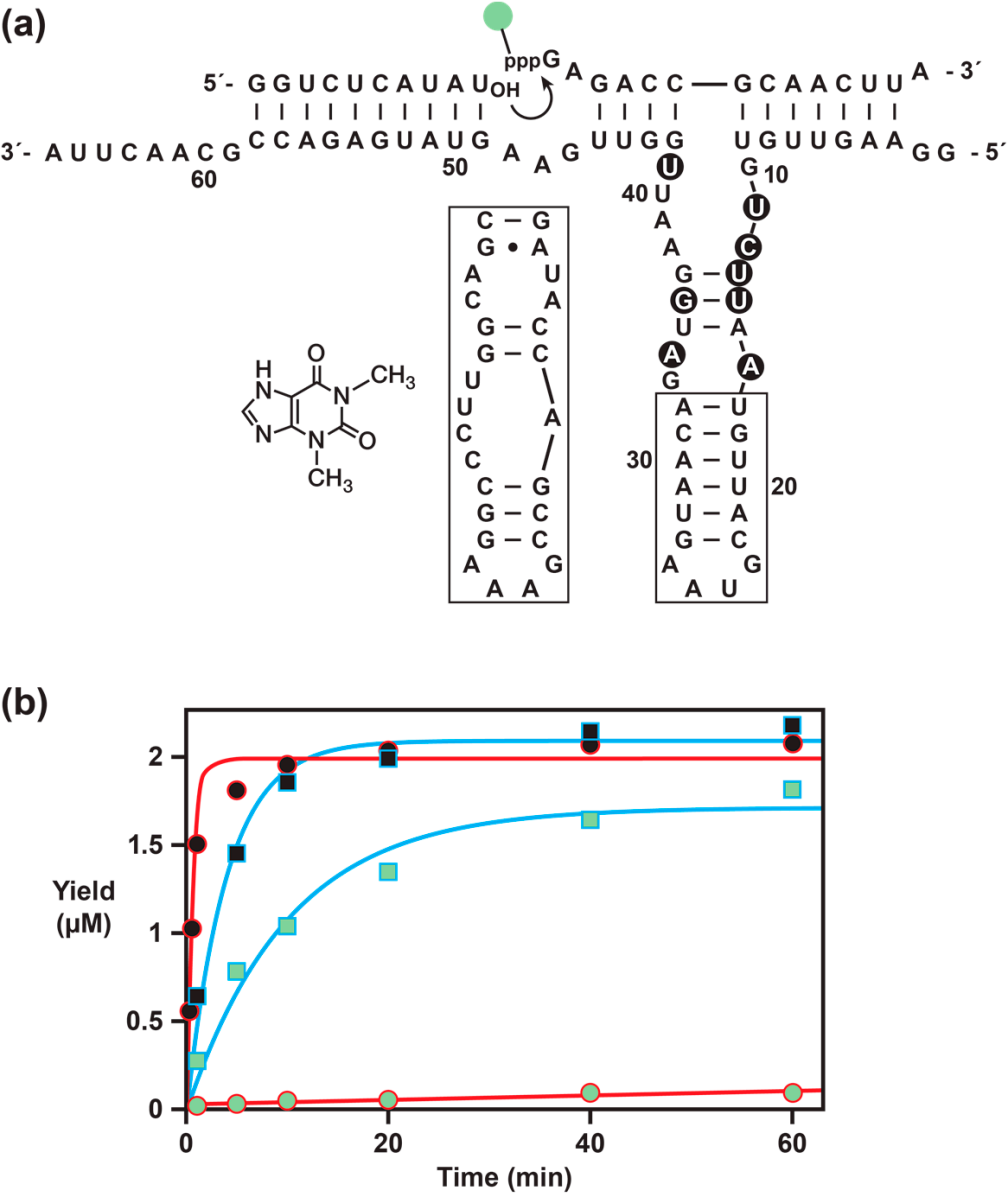
Composition and catalytic activity of the fluorophore-accommodating RNA enzyme. (**a**) Sequence and secondary structure of the selected enzyme, with mutations relative to the starting enzyme highlighted by black circles. The distal portion of the central stem-loop (boxed) can be replaced by an aptamer domain that binds its corresponding ligand. Shown at the left is the theophylline aptamer [12] and the chemical structure of theophylline; (**b**) Catalytic activity of the starting (red circles) and selected (blue squares) RNA enzymes with either unmodified substrate B (black symbols) or TMR-B (green symbols). Values for *k*_obs_ were obtained by fitting the data to the equation: yield = *a* × (1 − e*^−b^*^t^), where *a* is the maximum extent and *b* is the observed rate. The rate of reaction of the starting enzyme with TMR-B was determined by a linear fit of the data.

Individuals were cloned from the final selected population, sequenced, and tested for catalytic activity. The aligned sequences revealed a strong consensus with only slight variation. The dominant sequence, which contains eight mutations relative to the starting enzyme, is shown in Figure 2a. This molecule was prepared in the self-replication format and tested in a single-turnover ligation reaction employing 5 µM of a simplified form of substrate A having the sequence 5′-GGUCUCAUAU-3′, 2 µM of either unlabeled substrate B or TMR-B, 6 µM of E, and 25 mM of MgCl_2_, which were incubated at pH 8.5 and 42 °C. Under these conditions, the starting enzyme has a catalytic rate of 1.4 min^–1^ with the unmodified substrate B and only 0.0013 min^–1^ with TMR-B. In contrast, the selected enzyme has a catalytic rate of 0.25 min^–1^ with unmodified substrate B and 0.11 min^–1^ with TMR-B (Figure 2b).

The rate of the selected enzyme with the fluorophore-modified substrate is nearly 100-fold faster compared to that of the starting enzyme. This rate is similar when substrate B is instead labeled with Alexa Fluor 488 (data not shown). The selected enzyme is about 6-fold slower than the starting enzyme when reacting with the unmodified substrate. Thus, the selected enzyme has, to some extent, become specialized in reacting with the modified substrate.

### 2.3. Synthesis of the Fluorophore/Quencher Substrate

**Scheme 1:**
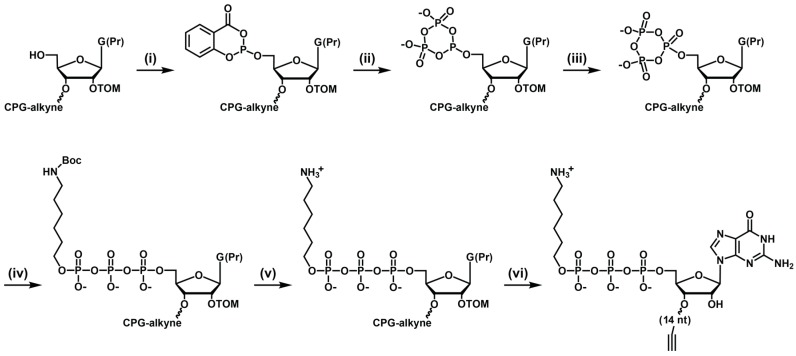
Synthesis of the TMR-B-Q substrate. (i) salicyl phosphorochloridite in 3:1 dioxane/pyridine, 15 min; (ii) TBAP in DMF for 20 min; washed with ACN/hexanes; (iii) 1 M of *t*-butyl peroxide in hexanes for 15 min; washed with ACN/hexanes; (iv) 3.5 M of 6-(Boc-amino)-1-hexanol in ACN for 72 h; washed with ACN; (v) 9:1 TFA/hexanes; washed with ACN; (vi) standard RNA deprotection.

Although the d-RNA form of TMR-B can be prepared by in vitro transcription and subsequent chemical modification, this is not possible for the corresponding l-RNA form, which must be prepared synthetically. The chemical synthesis of l-RNA oligonucleotides is straightforward, relying on commercially available l-RNA phosphoramidites. However, the l-RNA form of TMR-B requires installation of a 5′-triphosphate and subsequent modification of the γ-phosphate with the fluorescent label. In addition, for the real-time detection system, a quencher (Q) must be installed at the 3′-end of the substrate. Thus a synthetic scheme was devised to prepare the TMR-B-Q substrate (Scheme 1).

A 14-nucleotide d- or l-RNA, having the sequence 5′-GAGACCGCAACUUAU-3′, was prepared by solid-phase synthesis using 2′-*O*-triisopropylsilyloxymethyl (TOM) phosphoramidites on a CPG column derivatized with 2′-*O*-propargyl-uridine. Following removal of the 5′-terminal dimethoxytrityl group, but prior to further deprotection, the 5′-hydroxyl was phosphitylated using salicyl phosphorochloridite, reacted with tributylammonium pyrophosphate (TBAP) to form the cyclic trimetaphosphite, and oxidized with *t*-butyl peroxide to form the water-sensitive and reactive cyclic trimetaphosphate (Scheme 1, steps i–iii) [10,13,14,15]. Then, 6-(Boc-amino)-1-hexanol was coupled to the 5′-end of the substrate under strictly anhydrous conditions, using a modification of the Ludwig method (Scheme 1, step iv; J. Ludwig, personal communication). The 5′-terminal amine was deprotected using a 9:1 (*v*/*v*) solution of trifluoroacetic acid (TFA) in hexanes, the column was washed with acetonitrile (ACN), and the RNA was deprotected and purified using standard procedures (Scheme 1, steps v and vi). The free 5′-amine was labeled with TMR-NHS ester, then the product was purified by ethanol precipitation and dissolved in DMSO. The quencher TQ3 azide was conjugated to the terminal alkyne using click chemistry, and the final product was purified by PAGE.

The yield of synthetic TMR-B-Q was much greater than could be obtained by the in vitro transcription method. Thus, both the d-and l-RNA substrates were prepared in this manner. TQ3 was chosen as the quencher due to its close overlap with the emission spectrum of TMR. The quenching efficiency of TMR-B-Q relative to free TMR was determined to be >85%.

### 2.4. Detection of Ligation Activity in Real-Time

**Figure 3:**
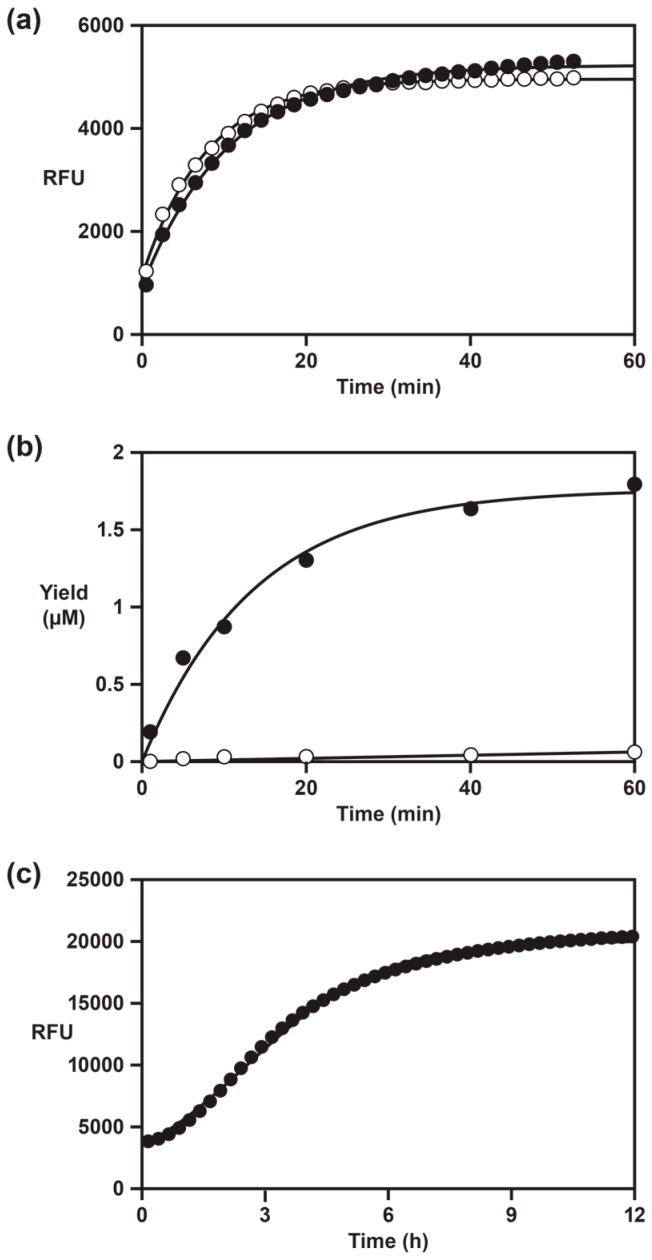
Functional properties of the selected RNA enzyme. (**a**) Real-time assay of RNA ligation, employing either d-RNA (black circles) or l-RNA (white circles). Values for *k*_obs_ were obtained as in Figure 2b, with the yield based on relative fluorescent units (RFUs); (**b**) Ligand-dependent activity of an RNA enzyme that contains a theophylline-binding aptamer domain, measured in either the presence (black circles) or absence (white circles) of theophylline; (**c**) Real-time monitoring of exponential amplification. The data were fit to the logistic growth equation: RFU = *a*/(1 + *b*e*^−c^*^t^), where *a* is the maximum extent, *b* is the degree of sigmoidicity, and *c* is the exponential growth rate.

The d- and l-RNA forms of the selected enzyme were tested in a real-time ligation assay. Both enzymes were prepared synthetically using 2′-*t*-butyldimethylsilyl (TBDMS) phosphoramidites. The d- and l-RNA substrates were prepared using 2′-TOM phosphoramidites. The ligation reaction employed the simplified form of substrate A and TMR-B-Q under the same conditions as above. Fluorescence was monitored in real time using a standard qPCR instrument, but maintaining a constant temperature of 42 °C. For both the d- and l-RNA forms of the reaction, there was a progressive increase in fluorescence, with observed rates of 0.10 and 0.13 min^−1^, respectively (Figure 3a). The slight difference in rates is attributable to a slight difference in the purity of the RNA phosphoramidites and resulting synthetic oligonucleotides [10].

### 2.5. Ligand-Dependent Activity

An aptamer that binds theophylline [12] was linked to the catalytic center of the selected RNA enzyme, replacing the central stem-loop of the enzyme by the ligand-binding domain of the aptamer (Figure 2a, boxed region). The length and sequence of the connecting stem were varied to maximize sensitivity of catalytic function to the presence of theophylline (data not shown). A stem consisting of a single G•C pair proved to be optimal in this regard.

The d-RNA form of the resulting aptazyme (E_theo_) was prepared by in vitro transcription and tested in a ligation reaction employing 5 µM of the simplified form of substrate A, 2 µM of TMR-B, 6 µM of E_theo_, either 0 or 5 mM of theophylline, and 25 mM of MgCl_2_, which were incubated at pH 8.5 and 42 °C (Figure 3b). In the presence of theophylline, the catalytic rate of the aptazyme is 0.074 min^−1^, whereas in the absence of ligand the rate is only 0.0008 min^−1^. Thus, the aptazyme exhibits substantial ligand dependence, with a ~100-fold rate enhancement in the presence compared with absence of ligand. The rate of the aptazyme in the presence of saturating ligand is similar to that of the ligand-independent form of the enzyme when reacting with the fluorophore-modified substrate. Based on previous studies with the theophylline-dependent aptazyme [3,4], subsaturating concentrations of ligand would result in a corresponding reduction of the catalytic rate.

### 2.6. Detection of Exponential Amplification in Real-Time

Self-replication requires that the product of ligation be identical to the enzyme. Thus, a variant form of substrate A was prepared that contains the same eight mutations present in the selected enzyme (Figure 2a). In order to achieve efficient exponential amplification with the selected enzyme, it was necessary to decrease the reaction temperature from 42 to 34 °C and to increase the concentration of MgCl_2_ from 25 to 100 mM. It has previously been shown that different forms of the self-replicating enzyme have different temperature optima and preferred MgCl_2_ concentration [8].

The self-replication reaction employed 20 µM of substrate A, 20 µM of TMR-B-Q, 50 nM of E, and 100 mM of MgCl_2_, which were incubated at pH 8.5 and 34 °C. The increase in fluorescence was monitored in real time, as described above. The data fit well to the logistical growth equation, exhibiting clear sigmoidal behavior that is characteristic of exponential amplification (Figure 3c). The exponential amplification rate is 0.61 h^−1^, which corresponds to a doubling time of 1.1 h. This is three-fold slower than the rate of the starting enzyme when operating with unmodified substrate B [6,7], but for the first time demonstrates the real-time fluorescence detection of a self-replicating RNA enzyme.

## 3. Discussion

The self-replicating RNA enzyme is the only known macromolecule that can produce additional copies of itself and undergo exponential amplification in a self-sustained manner. This enzyme has been used to study processes of molecular evolution relevant to the origins of life [6,8,16]. It also has been investigated for potential applications in molecular diagnostics and biosensing [3,4]. The latter make use of the ability of the enzyme to operate in a ligand-dependent manner, where the rate of exponential amplification is dependent on the concentration of the target ligand.

Following initial reports of ligand-dependent exponential amplification of RNA, three key shortcomings of the system were identified. First, because the enzyme and its substrate are composed of RNA, they are susceptible to degradation by ribonucleases that are present in biological samples. While it is possible to remove or inactivate these ribonucleases, such processing would affect other proteins in the sample that may be the target for detection. This shortcoming was addressed by preparing the enzyme and its substrates in the form of l-RNA, which is resistant to ribonuclease degradation [10]. A second shortcoming is that the original form of the self-replicating RNA enzyme has a doubling time of 23 min, requiring assay times of a few hours for the quantitative detection of a target ligand. Accordingly, an improved form of the enzyme was developed that has a doubling time of only 5 min and an exponential growth rate of 0.14 min^−1^ [8]. This rate is close to the limit of 0.21 min^–1^ imposed by the rate of product release of the E•E complex [7].

The present study sought to address the third shortcoming of the ligand-dependent exponential amplification system, which is the need to provide a convenient real-time assay, preferably one based on a simple fluorimetric readout. Methods based on FRET signaling and molecular beacons were explored, but suffered from high background and potential false positive signals. Instead, by linking the fluorescent reporter directly to the pyrophosphate leaving group, one obtains a direct measurement of the RNA ligation event that underlies exponential amplification. By also placing a fluorescence quencher at a suitable location within the substrate, the level of background fluorescence is very low. The key challenge was that, because the 5′-triphosphate is directly involved in the ligation reaction, it is generally intolerant of substitution. This limitation was overcome by using in vitro selection to obtain a variant enzyme that can operate efficiently when a fluorescent label is linked to the 5′-γ-phosphate of the substrate.

Exponential amplification of the selected enzyme with the labeled substrate is only three-fold slower than for the original self-replicating enzyme with the unmodified substrate. However, the rate-enhanced form of the enzyme described above is another five-fold faster when reacting with the unmodified substrate [8]. The constellation of eight mutations present in the fluorophore-accommodating substrate are not compatible with the six mutations present in the rate-enhanced enzyme. Thus, further efforts will be needed to combine the advantageous properties of both enzymes.

Regardless of the sequence of the enzyme, it can be readily prepared in the form of enantiomeric l-RNA having the same catalytic properties. This is why it was essential to prepare the fluorophore-modified substrate by chemical synthesis, making it available for use as either d- or l-RNA. Even for the d-RNA substrate, the synthetic approach is preferred because it does not require competition between γ-(6-aminohexyl)-GTP and GTP during in vitro transcription to install a derivatizable group on the 5′-γ-phosphate. Chemical synthesis also avoids the 5′-terminal sequence heterogeneity that commonly occurs during in vitro transcription [17,18].

A potential application of the fluorescent-signaling, self-replicating RNA enzyme is to monitor ligand-dependent exponential amplification in real time. The instrument used in this study was a standard qPCR device with 96-well format, although maintaining a constant reaction temperature rather than thermal cycling. The time required to reach an amplification threshold that is distinguishable from background is about 2–3 h (Figure 3c), which is longer than typically required for qPCR. The rate-enhanced self-replicating enzyme has an exponential growth rate comparable to PCR, but is not capable of direct fluorescent signaling. Despite the slower rate of the present system compared with PCR, it has the advantage of potentially being used to detect a wide variety of ligands, including small molecules, peptides, and proteins. As the inventory of known aptamers, including l-RNA aptamers, continues to expand, the opportunities to combine those reagents with the real-time exponential amplification system will expand accordingly.

## 4. Materials and Methods

### 4.1. Materials

Synthetic oligonucleotides were prepared by solid-phase synthesis using an Expedite 8909 DNA/RNA synthesizer. All nucleoside phosphoramidites and 2′-*O*-propargyl uridine 3′-Lcaa CPG were purchased from ChemGenes (Wilmington, MA, USA). Both T7 RNA polymerase and RNaseP M1 RNA were prepared as described previously [6]. Bovine pancreatic DNase I was purchased from Roche Life Science (Indianapolis, IN, USA), Superscript II RNase H^–^ reverse transcriptase and Alexa Fluor 488 NHS ester were from Thermo Fisher (Carlsbad, CA, USA), and yeast poly(A) polymerase was from Affymetrix (Santa Clara, CA, USA). Tributylammonium pyrophosphate, 6-(Boc-amino)-1-hexanol, TMR-NHS ester, biotin-PEG4-alkyne, NTPs, dNTPs, and theophylline were purchased from Sigma-Aldrich (St. Louis, MO, USA), γ-(6-aminohexyl)-GTP was from Jena Bioscience (Jena, Germany), 2′-azido,2′-deoxyadenosine 5′-triphosphate was from TriLink Biotechnologies (San Diego, CA, USA), and Tide Quencher™ 3 (TQ3) azide was from AAT Bioquest (Sunnyvale, CA, USA).

The TMR-B-biotin substrate was prepared by in vitro transcription of an extra-length RNA, which was cleaved using *E. coli* RNase P M1 RNA and an external guide RNA to yield RNA with a homogeneous end and free 2′- and 3′-hydroxyls [3,8,19]. The transcription mixture contained 2 mM of GTP and 5 mM each of γ-(6-aminohexyl)-GTP, ATP, and CTP. The external guide RNA had the sequence 5′-GGAGUAAGUUGCGGUCUCACCA-3′ (region of hybridization underlined). Following RNA cleavage, the RNA was extended by a single 2′-azido,2′-deoxyadenosine residue using yeast poly(A) polymerase, then coupled to biotin-PEG4-alkyne, as described previously [8].

In vitro transcribed RNA enzymes were prepared by run-off transcription, as described previously [4,6]. Double-stranded DNA templates encoding the enzyme were prepared by cross-extension of two fully-complementary synthetic oligodeoxynucleotides. For the selected enzyme the sense strand had the sequence 5′-GGCTAATACGACTCACTATAGGAAGTTGTGTCTTAATGTTACGTAAGTAACAGATGGAATTGGTTGAAGTATGAGACCGCAACTTA-3′; for E_theo_, the sense strand had the sequence 5′-GGCTAATACGACTCACTATAGGAAGTTGTGTCTTAA**GATACCAGCCGAAAGGCCCTTGGCAGC**GATGGAATTGGTTGAAGTATGAGACCGCAACTTA-3′ (T7 RNA polymerase promoter sequence underlined; aptamer domain in bold).

In vitro transcribed substrate A containing the eight mutations present in the selected enzyme was prepared by first generating the enzyme, as described above. Then, the B-substrate portion was removed using M1 RNA and the same external guide RNA described above.

Synthetic d- and l-RNA enzymes and both the full-length and simplified forms of substrate A were prepared by automated solid-phase synthesis using 2′-TBDMS phosphoramidites. The RNAs were deprotected using a 1:1 mixture of aqueous ammonia and methylamine at 65 °C for 10 min, then lyophilized. Then, the 2′-TBDMS group was removed by incubation with tetrabutylammonium fluoride for 16 h. The resulting materials were mixed with an equal volume of 1 M Tris (pH 7.5) and desalted, and the RNAs were purified by PAGE.

### 4.2. Synthesis of TMR-B and TMR-B-Q Substrates

Both the d- and l-RNA forms of substrate B were prepared by automated solid-phase synthesis using 2′-TOM phosphoramidites. For substrates containing the TQ3 quencher, synthesis was carried out on a 2′-*O*-propargyl uridine CPG column. The substrates were 5′-triphosphorylated on the column, based on a modification of previous methods [10,14] (Scheme 1). Following removal of the 5′-dimethoxytrityl protecting group, the column was removed from the DNA/RNA synthesizer and connected at each end to a syringe, one fitted with a 3-way stopcock linked to both an argon source and an injection port, and the other fitted with a plunger to allow mixing and withdrawal of waste material. The 5′-hydroxyl was phosphitylated with salicyl phosphorochloridite in 3:1 dioxane/pyridine for 15 min, this solution having been prepared just prior to use by dissolving 10 mg of dry salicyl phosphorochloridite in 100 µL of dioxane/pyridine. The resulting material is extremely sensitive to water and must be maintained under dry argon. It was converted to the cyclic trimetaphosphite using 250 µL of dry tributylammonium pyrophosphate in DMF for 20 min, then washed twice with 500 µL of dry ACN/hexanes. Then, the cyclic trimetaphosphite was oxidized to the cyclic trimetaphosphate using 250 µL of dry 1 M *t*-butyl peroxide in hexanes for 15 min. The resulting material was washed with 500 µL of dry ACN and allowed to react with dry 3.5 M of 6-(Boc-amino)-1-hexanol in ACN for 72 h [15], and then again washed with dry ACN. The 5′-terminal amine was deprotected using 9:1 TFA/hexanes, and the column was then washed with 100 mM of Tris (pH 7.5), washed with ACN, and dried under argon.

The oligonucleotide was deprotected as described above, and the 5′-amine was then labeled with TMR-*N*-hydroxysuccinimidyl ester according to the manufacturer’s directions, followed by ethanol precipitation. For the TMR-B-Q substrate, the 3′-terminal alkyne was coupled with TQ3 azide in a click reaction, employing 25 µM of RNA, 150 µM of TQ3 azide, 0.4 mM of Cu(II)SO_4_, 2 mM of Tris(3-hydroxypropyltriazolylmethyl)amine, 10 mM of sodium ascorbate, 50 mM of Na_2_HPO_4_ (pH 7.4), and 10% dimethyl sulfoxide, which was incubated at 23 °C for 2 h. The resulting materials were purified by PAGE and ethanol precipitation.

### 4.3. In Vitro Selection

The in vitro selection procedure was based on a previously reported method [8]. The starting DNA library had the sequence 5′-GGCTAATACGACTCACTATAGGAGCGAGAACGTTGT-N_7_-TGTTACGTAAGTAACA-N_9_-GTTGAAGTATGAGACCGGAAGGTTGAAGTATGAGACCGCAACGTA (T7 RNA polymerase promoter sequence underlined; N indicates nucleotides that were fully randomized). The DNA was made double-stranded by extending a primer having the sequence 5′-TACGTTGCGGTCTCATACTTCAACCTTCCGGTCTCATACTTCAACC-3′; then, 1.7 × 10^13^ DNA molecules (282 pmol) were used to transcribe the corresponding pool of RNAs. These were cleaved using M1 RNA and an external guide RNA having the same sequence as described above for the processing of substrate B. Following purification by PAGE, 6.0 × 10^12^ RNA molecules (100 pmol) were mixed with TMR-B-biotin substrate and 50 mM of EPPS (pH 8.5), heated to 70 °C for 2 min, and then cooled to 4 °C over 3 min. The mixture was then heated to 42 °C, and the ligation reaction was initiated by adding MgCl_2_ and then quenched by adding 50 mM of EDTA. During the first round of selection the reaction mixture contained 2 µM of TMR-B-biotin, 1 µM of E-A, and 25 mM of MgCl_2_, and the reaction time was 1 h. During rounds 2 and 3, the concentration of reactants was the same, and reaction time was reduced to 7.5 min in round 2 and to 30 s in round 3. During rounds 4 and 5, the reaction mixture contained 50 nM of TMR-B-biotin and 50 nM of E-A, the concentration of MgCl_2_ was 25 mM in round 4 and 5 mM in round 5, and the reaction time was 30 s in round 4 and 15 s in round 5.

The ligated products were captured on a streptavidin-agarose resin and washed successively with the following: 10 mL of a solution containing 100 mM of NaCl, 1 mM of EDTA, and 50 mM of Tris (pH 7.5); 20 mL of a solution containing 1 M of NaCl, 1 mM of EDTA, 0.1% Triton X-100, and 50 mM of Tris (pH 7.5); 10 mL of a solution containing 100 mM of NaCl, 1 mM of EDTA, and 50 mM of Tris (pH 7.5); and 20 mL of a solution containing 1 mM of EDTA and 10 mM of Tris (pH 7.5). The ligated RNA was reverse transcribed on the resin by extending a primer having the sequence 5′-TAAGTTGCGGTCTC-3′. The DNA (as an agarose slurry) was then PCR amplified using the same reverse primer and a forward primer having the sequence 5′-GGCTAATACGACTCACTATAGGAGCGAGAACGTTGT-3′ (T7 RNA polymerase promoter sequence underlined). The amplified DNA was in vitro transcribed, and the resulting RNA was purified by PAGE and then used to initiate the next round of selection.

### 4.4. Ligation Assay

Ligation was carried out by first mixing 5 µM of substrate A, 2 µM of substrate B (unlabeled B, TMR-B, or TMR-B-Q), 6 µM of enzyme (E or E_theo_), and 50 mM of EPPS (pH 8.5), which were heated at 70 °C for 2 min and then cooled to 4 °C over 3 min. The mixture was then heated to 42 °C, and the reaction was initiated by adding MgCl_2_ to a final concentration of 25 mM. Aliquots were taken from the mixture at various times. The products were analyzed by PAGE, and imaging fluorescence with a BioRad Pharos FXPlus Molecular Imager. For the real-time assays, the reaction mixture was monitored continuously using a BioRad CFX-96 Real-Time PCR Detection System.

### 4.5. Exponential Amplification Assay

Amplification was carried out by first mixing 20 µM of substrate A, 20 µM of TMR-B-Q, 50 nM of E, and 50 mM of EPPS (pH 8.5), which were heated to 70 °C for 2 min and then cooled to 4 °C over 3 min. The mixture was then heated to 42 °C, and the reaction was initiated by adding MgCl_2_ to a final concentration of 100 mM. The course of the reaction was monitored using a BioRad CFX-96 Real-Time PCR Detection System.

## 5. Conclusions

A self-replicating RNA enzyme, which can undergo exponential amplification in a ligand-dependent manner, was modified to generate a fluorescent signal throughout the course of amplification. This modification entailed both derivatization of the leaving group with a fluorescent reporter and optimization of the enzyme to accommodate that reporter. The result is a novel way to measure the concentration of various target ligands with high sensitivity and specificity.
